# Autonomic function in a prevalent Tanzanian population with Parkinson’s disease and its relationship to disease duration and 5-year mortality

**DOI:** 10.1186/1756-0500-6-535

**Published:** 2013-12-17

**Authors:** Eric Aris, Catherine L Dotchin, William K Gray, Richard W Walker

**Affiliations:** 1 Department of Internal Medicine, Muhimbili University College of Health Sciences, Dar es Salaam, Tanzania; 2 Institute for Ageing and Health, Campus for Ageing and Vitality, Newcastle University, Newcastle upon Tyne, UK; 3 Department of Medicine, North Tyneside District General Hospital, Rake Lane, North Shields, Tyne and Wear, UK; 4 Institute of Health and Society, Newcastle University, Newcastle upon Tyne, UK

**Keywords:** Parkinson’s disease, Africa, Autonomic function, Tanzania

## Abstract

**Background:**

Autonomic dysfunction is common in patients with Parkinson’s disease (PD). We report autonomic function test results in a prevalent, largely untreated, Tanzanian population of PD patients, at different disease stages and investigate the relationship between autonomic dysfunction and mortality.

**Methods:**

Ewing’s battery of autonomic tests was carried out on a prevalent population of PD patients living in the rural Hai district of Tanzania. Where possible, all four tests were performed in the patient’s home. The main outcome of interest was the presence of abnormalities of sympathetic or parasympathetic function. Information on medications used and other co-morbidities was recorded.

**Results:**

Autonomic function tests were recorded for 29 subjects, of whom 3 were on medication at the time of assessment. Of the 26 unmedicated patients, 14 (53.8%) had at least one abnormal test result for autonomic function, of whom only 3 (21.4%) were in late stage disease (Hoehn and Yahr stage IV or V), compared to 7 (58.3%) of 12 with normal autonomic function tests in late stage disease. Ten subjects had died at 5-year follow-up, but there was no association between mortality and autonomic function test abnormalities.

**Conclusions:**

In unmedicated subjects, many patients in late stage disease had relatively preserved autonomic function, compared to those in early stage disease. In people with PD who are taking medication, it may be that when autonomic dysfunction presents in late stage disease it is often due to side effects of medication rather than the disease itself.

## Background

Autonomic symptoms are frequently seen in patients with Parkinson’s disease (PD). These include urinary symptoms, sweating disturbance, constipation, sexual dysfunction and blurred vision [1]. Dysregulation of parasympathetic cardiovascular control mechanisms is a frequent complication of early stage PD [2,3]. Such dysregulation can manifest itself as a range of measurable changes in cardiac function such as heart rate variability and orthostatic hypotension (OH). OH is thought to be present in around 50% of people with PD and can be particularly distressing, with an increased likelihood of serious injury from falls [2,4].

It is thought that these autonomic symptoms may be linked to PD medication as well as neurodegenerative disease related changes in the function of the autonomic nervous system [5]. Therefore, data from unmedicated populations are of particular interest. The primary aim of this study was to investigate autonomic dysfunction within a prevalent, largely untreated, PD population from rural Tanzania [6]. Secondary aims were to consider associations with demographic and clinical data and five-year mortality.

## Methods

Ethical approval for the study was obtained through the National Institute of Medical Research, Tanzania and the Newcastle and North Tyneside Ethics Committee in the UK. Patient information sheets and consent forms were translated into Swahili by a Tanzanian PD nurse specialist.

All patients were identified from a previously reported prevalence study [6]. After obtaining informed consent, all patients were assessed either in their own homes or, if possible, a local health clinic by a research doctor (C.D.). Translation was through local assistant medical officers, census enumerators and a PD nurse specialist. PD was diagnosed according to the UK Brain Bank criteria [7]. The patients were videoed and reviewed by a movement disorders specialist (R.W.) to confirm the diagnosis.

### Autonomic function tests

The autonomic function tests were based on Ewing’s battery of four tests [8]. The tests were all carried out by the research doctor who had been trained in PD and autonomic function testing.

### Supine and standing BP: sympathetic function

Baseline systolic and diastolic BP was measured in a supine position after five minutes resting quietly. Three measurements were taken at one-minute intervals and the mean calculated. The patient was then asked to stand up. Three standing BP results were recorded; immediately on standing, after 30 seconds and after one minute standing up. The lowest systolic BP value of the three was recorded. A decrease in systolic BP, from the mean supine BP, of greater than 20 mmHg was recorded as an abnormal result.

### BP response to hand grip: sympathetic function

BP response to hand grip was measured with a hand-held sphygmomanometer. Patients were given the instruction “to squeeze the machine as tightly as possible” with their dominant hand to determine their maximum possible power, and then to hold it at one third of their maximum total power for as long as possible up to five minutes. During this period, BP was measured three times at one minute intervals and the highest result noted. The mean resting diastolic BP was subtracted from the highest diastolic BP during handgrip and the result recorded. Failure to increase diastolic BP by more than 20 mmHg during the prolonged handgrip was recorded as abnormal.

### Heart rate response to standing: Parasympathetic function

To record heart rate response to standing, patients were asked to lie down quietly for at least 5 minutes. The patient had the 12 lead ECG machine attached with continuous rhythm strip recording. When the patient stood up a point was marked on the ECG trace and recording continued for a further 30 beats. R-R variability was measured as the ratio of the longest R-R interval (at or around 30 beats) compared to the shortest R-R interval (at or around 15 beats). This ratio (30:15 ratio) was then classified as being abnormal if ≤ 1.00.

### Heart rate variability to deep breathing: parasympathetic function

Respiratory heart rate variability was measured with a 12 lead ECG machine put on continuous rhythm strip recording, with the patient lying supine and relaxed breathing in and out slowly at a rate of six breaths per minute for one minute.

### Other data collected

Demographic data (age and gender) were also collected. The Unified Parkinson’s Disease Rating Scale (UPDRS) [9] and the 39-item Parkinson’s Disease Questionnaire (PDQ-39), measuring quality of life, were administered [10].

### Five-year follow-up

All patients were followed-up at five years after initial assessment. Details of any patient who had died during this period were recorded.

### Statistical analysis

The data were quantitative in nature and collected at a nominal, ordinal and interval/ratio level. They were analyzed using standard statistical software, *PASW-18 for windows* (PASW, Chicago, IL, USA). Two-tailed tests were used throughout, the significance level was set at α = 0.05.

## Results

Thirty-two cases (22 males, 68.8%) of PD were identified by the prevalence study [6]. The mean age was 73.8 years (standard deviation 12.90). For three cases (2 males), none of the four tests of autonomic function could be completed successfully and they are excluded from the current study. A further three cases were on medication at the time of assessment; data for these cases are analysed separately. Two subjects were taking benzhexol and one was taking sinemet. Only two other cases had received any type of anti-parkinsonian medication in the past though both had not taken medication for some time.

For the test of heart rate variability to deep breathing, many of the patients, not just those with cognitive impairment, found it difficult to understand the instructions for breathing slowly without trying to breathe as deeply as they could or hold their breath. It took several attempts to get the patients to comply with this, and the target of 6 breaths per minute was not always achieved. It was felt these data were unreliable and they were not utilised.

### Untreated subjects (n = 26)

Results of autonomic function tests are shown in Table 1. For the test of heart rate response to standing, 9 patients were unable or unwilling to stand whilst connected to the ECG machine for long enough for a recording to be made. For the test of BP response to standing, some patients were unable to stand for long enough for 3 complete BP recordings to be completed. In these cases, if the first recording was normal then this result was accepted and no further recording made, thus data for all 26 patients are presented. For the tests of BP response to hand grip, 4 patients were unable to grip for long enough to make a satisfactory recording.

**Table 1:** Results of autonomic function tests and outcomes for treated and untreated patients

**Untreated patients (n = 26)**	
Heart rate response to standing	4 (23.5%) abnormal, 13 (76.5%) normal, 9 assessment not completed
BP response to hand grip	11 (50%) abnormal, 11 (50%) normal, 4 assessment not completed
BP response to standing	1 (3.8%) abnormal, 25 (96.2%) normal
One or more abnormal tests	14 (53.8%)
Died by 5-year follow-up	10 (38.5%)
**Treated patients (n = 3)**	
Heart rate response to standing	1 (33.3%) abnormal, 2 (66.7%) normal
BP response to hand grip	2 (66.7%) abnormal, 1 (33.3%) normal
BP response to standing	1 (33.3%) abnormal, 2 (66.7%) normal
One or more abnormal tests	2 (66.7%)
Died by 5-year follow-up	2 (66.7%)

BP = blood pressure.

Of the 26 untreated patients who had at least one autonomic test performed, 14 (53.8%) had one or more abnormal test result. Interestingly, only 3 (21.4%) of those with abnormal results were in late stage disease (Hoehn and Yahr stage IV or V), compared to 7 (58.3%) of the 12 with normal test results. This difference was not significant (χ^2^(1) = 3.718, p = 0.054), although this is likely to be in part due to the small numbers involved, representing a type II error. However, having at least one abnormal autonomic function test result was associated with greater quality of life by PDQ-39 (U = 21.5, z = −3.216, p = 0.001), greater physical function (U = 38.0, z = −2.377, p = 0.017) and improved UPDRS score (U = 31.5, z = −2.493, p = 0.013). All three of these variables are also markers for disease severity.

At 5-year follow up, 10 (38.5%) of the 26 untreated subjects had died. There was no association between having at least one abnormal autonomic function test and age, sex or 5-year mortality.

### Treated subjects (n = 3)

Of the three treated subjects, one had abnormal heart rate response to standing, two had abnormal BP response to hand grip and one had abnormal BP response to standing (see Table 1). Two of the three subjects had at least one abnormal test of autonomic function and two were in late stage disease based on Hoehn and Yahr score, both of whom had died at 5-year follow-up.

## Discussion

### Rates of autonomic dysfunction

Autonomic function test abnormalities were frequently seen in this group of patients. The most common abnormality seen was heart rate variability to hand grip, which was present in over half of those tested. Interestingly, the least common abnormality was OH which would be the baseline test performed in most medical clinics. Our estimates of the prevalence of autonomic dysfunction are similar to those reported in other populations in high-income countries [11]. Higher rates have been reported in another study, with 16 of 20 patients of stage I or II disease displaying signs of autonomic dysfunction, with cardiac symptoms particularly common [3]. In a Nigerian study, Okubadejo et al [12] reported just over half of patients with PD aged 65 years or over had parasympathetic dysfunction.

### Association between autonomic dysfunction and disease severity

The presence of autonomic dysfunction was associated with less advanced disease, less disability and greater quality of life. Although the reasons for this are far from clear, it appears that, in our cohort autonomic dysfunction is more common in early stage disease. Indeed, it is remarkable that in untreated patients, over two-thirds at Hoehn and Yahr stage IV or V had no autonomic dysfunction. It has previously been suggested that prominent autonomic features early in the course of the disease are a worrying or atypical feature, favouring multiple system atrophy as the underlying diagnosis [5]. There is evidence that autonomic involvement can occur in the early stages of PD; in one study, 38% of patients with abnormal autonomic function tests were in the first 5 years of their disease [11].

Previous research has suggested no significant association between autonomic dysfunction and mortality in people with PD [13]. In our largely untreated population, the lack of dysfunction in late stage disease may partly explain this observation.

### Appropriateness of the tests performed and limitations

Our figures for autonomic dysfunction are likely to be an under-estimate. Only two of the four tests could be performed satisfactorily on three-quarters of the cohort. If more patients had been able to undergo testing it is likely that our estimate would have been higher. Difficulties in performing autonomic tests have been reported in other centres too, even when conducting tests in a hospital setting. Magerkurth et al [11] documented that 20% of their patients could not satisfactorily perform the Valsalva manoeuvre with cardiovascular monitoring.

The test of R-R variability could not be performed on some patients due to frailty, a problem that could have arisen in any community-based PD cohort and was not unique to SSA. The fourth test, R-R variation with deep breathing would have been possible to test, but patients did not fully understand the instructions and in many cases the patient was breathing more quickly than the test required. In the study by Magerkurth et al [11] only 1 patient out of 141 tested had any abnormality in heart rate variability on deep breathing even though 56% reported one or more autonomic symptoms, so this may not be the most sensitive test for detecting autonomic dysfunction in PD. The autonomic tests reported measure cardiovascular reflexes and we did not test bladder function or sweating. In this setting, asking personal questions about continence was often difficult and questions on sexual function were not discussed, so we have no data on the frequency of erectile dysfunction in men. As the patients were visited at home, often many of the family, neighbours and local children had come to see what was going on. It was too dark inside the mud huts to conduct the interview indoors, so interviews were conducted in the communal courtyard, and it was not appropriate to ask intimate questions in that location.

Finally, we acknowledge that the small number of patients involved in our study, limits the extent to which conclusions can be drawn regarding their significance. However, in this resource limited setting, where diagnosis is rare, recruiting large numbers of PD patients is difficult. This is reflected in the extremely limited amount of previous data regarding PD in SSA.

## Conclusions

No previously published study has investigated a predominantly unmedicated population with such a wide variation of disease stage. Autonomic function test abnormalities were surprisingly uncommon in late stage disease. This may suggest that when such abnormalities are seen in late stage disease, there may be a significant contribution from anti-parkinsonian medication.

## Competing interests

The authors declare that they have no competing interests.

## Authors’ contributions

EA, CD and RW were responsible for designing the study and developing the study protocol. EA and CD collected the data. WKG and CD analysed and interpreted the data and produced a first draft of the manuscript. All authors critically revised and approved the final manuscript for submission.
